# Incidence of Norovirus-Associated Medical Encounters among Active Duty United States Military Personnel and Their Dependents

**DOI:** 10.1371/journal.pone.0148505

**Published:** 2016-04-26

**Authors:** Brian Rha, Benjamin A. Lopman, Ashley N. Alcala, Mark S. Riddle, Chad K. Porter

**Affiliations:** 1 Division of Viral Diseases, National Center for Immunization and Respiratory Diseases, Centers for Disease Control and Prevention, Atlanta, Georgia, United States of America; 2 Epidemic Intelligence Service, Centers for Disease Control and Prevention, Atlanta, Georgia, United States of America; 3 Naval Medical Research Center, Silver Spring, MD, United States of America; Iran University of Medical Sciences, ISLAMIC REPUBLIC OF IRAN

## Abstract

**Background:**

Norovirus is a leading cause of gastroenteritis episodes and outbreaks in US military deployments, but estimates of endemic disease burden among military personnel in garrison are lacking.

**Methods:**

Diagnostic codes from gastroenteritis-associated medical encounters of active duty military personnel and their beneficiaries from July 1998–June 2011 were obtained from the Armed Forces Health Surveillance Center. Using time-series regression models, cause-unspecified encounters were modeled as a function of encounters for specific enteropathogens. Model residuals (representing unexplained encounters) were used to estimate norovirus-attributable medical encounters. Incidence rates were calculated using population data for both active duty and beneficiary populations.

**Results:**

The estimated annual mean rate of norovirus-associated medically-attended visits among active duty personnel and their beneficiaries was 292 (95% CI: 258 to 326) and 93 (95% CI: 80 to 105) encounters per 10,000 persons, respectively. Rates were highest among beneficiaries <5 years of age with a median annual rate of 435 (range: 318 to 646) encounters per 10,000 children. Norovirus was estimated to cause 31% and 27% of all-cause gastroenteritis encounters in the active duty and beneficiary populations, respectively, with over 60% occurring between November and April. There was no evidence of any lag effect where norovirus disease occurred in one population before the other, or in one beneficiary age group before the others.

**Conclusions:**

Norovirus is a major cause of medically-attended gastroenteritis among non-deployed US military active duty members as well as in their beneficiaries.

## Introduction

Noroviruses are a major cause of epidemic and sporadic gastroenteritis worldwide [1–4]. In the United States, norovirus is estimated to cause 19–21 million episodes of gastroenteritis, 1.7–1.9 million medical outpatient visits, 400,000 emergency department visits, 56,000–71,000 hospitalizations, and 570–800 deaths annually [2, 5–8]. The total annual health care and social costs for norovirus disease in the US have been estimated at $5.5 billion [9]. However, the lack of a widely used, rapid and sensitive clinical assay has made detailed characterizations of norovirus activity, impact, and burden challenging in the range of settings where they cause disease.

Norovirus outbreaks are common among groups inhabiting relatively confined spaces such as nursing homes, daycares, cruise liners, and military settings such as barracks and ships. In military populations, numerous outbreaks of norovirus gastroenteritis have been documented among troops deployed in support of Operations Iraqi and Enduring Freedom, on ships, as well as among recruit training populations [10]. However, estimates of disease incidence and burden have not been published for this population.

Efforts to characterize the norovirus disease burden in military settings face the same challenges seen in civilian settings, such that methodological approaches developed for civilian populations can be used to study the US military and their beneficiary populations [11]. Given the potential health burden of norovirus in military populations [10], impact on mission-readiness and training [12], and long-term sequelae [13, 14], efforts to better understand disease incidence are warranted, especially in the context of vaccine development.

This study utilized a time-series regression approach to estimate the incidence of norovirus-attributable disease based on medical encounter data from US Department of Defense (DoD) populations seeking care for acute gastroenteritis. We generated incidence estimates for both active duty populations and their dependent beneficiaries (e.g. spouses and children).

## Methods

### Data Sources

Our study populations were all active duty (AD) military personnel and their dependent beneficiaries (DB) who sought medical care in weeks with start dates between July 5, 1998 to June 26, 2011 or July 1, 2001 to June 26, 2011, respectively. Medical claims data including encounters at DoD and non-DoD medical treatment facilities from the Standard Ambulatory Data Record (SADR), Standard Inpatient Data Record (SIDR), Health Care Service Records (HCSR), TRICARE Encounter Data (TED), and the Tri-Service Reportable Events Database were supplied by the Armed Forces Health Surveillance Center (AFHSC) [15, 16]. The study protocol was approved by the Naval Medical Research Center Institutional Review Board in compliance with all applicable Federal regulations governing the protection of human subjects; informed consent was not required for our analyses. To prevent duplication of records, reportable events were compared with documented medical encounters in the SIDR and SADR databases. If an event was documented in both datasets within 7 days, only a single observation was retained.

We extracted data for all gastroenteritis-associated medical encounters as identified by diagnosis codes (*International Classification of Diseases*, *Ninth Revision*, *Clinical Modification* [ICD9-CM]; Table 1) recorded in the first or second diagnostic position. Data were organized into weekly counts of bacterial, viral, protozoal and cause-unspecified encounters. Total monthly AD and yearly DB enrollment data were obtained from the Defense Manpower Data Center (DMDC) and used to estimate population-based rates of inpatient and outpatient encounters.

**Table 1:** Pathogen-based categories of diagnostic codes used to identify gastroenteritis-associated medical encounters, supplied by the United States Armed Forces Health Surveillance Center, July 1998–June 2011.

Pathogen Category and Diagnoses	ICD-9-CM* Code(s)
***Cause unspecified***	
Infectious colitis, enteritis, and gastroenteritis	009.0
Colitis, enteritis, and gastroenteritis of presumed infectious origin	009.1
Infectious diarrhea	009.2
Diarrhea of presumed infectious origin	009.3
Presumed noninfectious	558.1–558.9
Symptom: Diarrhea NOS	787.91
Other viral enteritis	008.64–008.69
Intestinal infection due to other organism not elsewhere classified	008.8
***Rotavirus***	008.61
***Adenovirus***	008.62
***Norovirus (Norwalk)***	008.63
***Bacterial***	
Cholera	001 (all subgroups)
Typhoid/Paratyphoid	002.0–002.9
Salmonella	003.0–003.9
Shigella	004 (all subgroups)
*Vibrio parahaemolyticus*	005.4
*Escherichia coli*	008.0
Campylobacter	008.43
Yersinia	008.44
*Clostridium difficile*	008.45
Other/unspecified bacteria	008.1–008.5
Other bacterial food poisoning	005.0–005.9
***Protozoal***	
Ameba	006.0–006.2; 006.9
Other protozoal	007 (all subgroups)

*International Classification of Diseases, Ninth Revision, Clinical Modification

### Statistical Analyses

Because norovirus is rarely laboratory-confirmed and is underrepresented in ICD9-CM-coded diagnoses [17], our goal was to estimate norovirus-attributable medical encounters among the cause-unspecified gastroenteritis-associated encounters for both AD and DB populations. We used a previously developed [6–8, 18–20] time-series regression approach to model the weekly number of cause-unspecified codes as a function of cause-specified codes, assuming counts of cause-unspecified codes follow a Poisson distribution. Cause-specified encounters were grouped by pathogen-based categories, including rotavirus, adenovirus, norovirus, bacterial, and protozoal-coded encounters (Table 1). We calculated the number of unspecified gastroenteritis encounters by summing encounters coded as unspecified gastroenteritis/diarrhea and other viral gastroenteritis. Similar to previous approaches [6, 7], presumed noninfectious coded encounters were included as unspecified due to the likelihood that episodes of gastroenteritis without a known etiology are often misclassified as such. We assumed that among the cause-unspecified gastroenteritis/diarrhea encounters, weekly encounters due to a specific pathogen group were proportional to the encounters coded for that pathogen group in that week.

Beneficiaries were modeled separately by age group (all ages, 0–4, 5–17, 18–39, 40–64 years). The ≥65-year DB age group was included only in the model for “all ages” due to small numbers (<0.1% of DB encounters). Active duty members were initially modeled as one combined age group. The model can be expressed by the formula:

$$E\left(CU_{x,y}\right)=\alpha +\left(\beta_{1}\times Bact_{x,y}\right)+\left(\beta_{2}\times Proto_{x,y}\right)+\left(\beta_{3}\times Rota_{x,y}\right)+\left(\gamma \times Time_{y}\right)+\left(\delta \times Fed\right)$$

where *CU*, *Bact*, *Proto*, and *Rota* are the counts of diagnostic codes in the cause-unspecified, bacterial, protozoal, and rotavirus categories, respectively (Table 1), for age group *x* and week *y*. Adenovirus-specific diagnostic code counts were not included in the model due to small numbers that showed no clear seasonal pattern. *α* represents the number of background, aseasonal encounters not explained by any pathogen-coded categories, *Time* is a sequential variable for the week of the coded encounter included to account for any secular trends, and *Fed* is the categorical variable accounting for weeks that included a federal holiday, accounting for decreased medical encounters in these weeks, similar to an approach taken previously [21, 22]. Poisson models were fitted using robust standard errors to account for overdispersion in the outcome variable.

The number of medical encounters not explained by the model (i.e., the model residuals) exhibited winter seasonality consistent with known norovirus epidemiology. These residuals were used to estimate the number of norovirus encounters by taking the difference between the weekly residual and the minimum weekly residual for a given seasonal year (July through June of the following calendar year). This assumed that any seasonality in unspecified visits not explained by the model were due to norovirus, and that no norovirus visits took place during the week with the minimum residual for each seasonal year. Rates of norovirus-associated medical encounters were estimated by seasonal year using annual enrollment data and expressed as the number of encounters per 10,000 persons. Coded norovirus-encounters, although captured in the data (Table 1), were not included in the estimated norovirus-attributable counts due to small numbers (mean of <40 encounters per seasonal year in both AD and DB populations; Table 2).

**Table 2:** Coded medical encounters by population and cause category.

Cause	Encounters by Active Duty Members (July 1998–June 2011)			Encounters by Dependent Beneficiaries (July 2001–June 2011)		
	N	Seasonal Year* Mean No. (%)	Encounters per 10,000 Persons	N	Seasonal Year* Mean No. (%)	Encounters per 10,000 Persons
Cause unspecified	1,732,903	133,300 (98.5)	936.4	1,764,784	176478.4 (98.2)	335.1
Bacterial	21,955	1,689 (1.2)	11.9	20,337	2033.7 (1.1)	3.9
Protozoal	2,502	192 (0.1)	1.4	3,078	307.8 (0.2)	0.6
Rotavirus	303	23 (<0.1)	0.2	8,060	806 (0.4)	1.5
Adenovirus	464	36 (<0.1)	0.3	539	53.9 (<0.1)	0.1
Norovirus	435	33 (<0.1)	0.2	342	34.2 (<0.1)	0.1
All-cause gastroenteritis	1,758,562	135,274	950.2	1,797,140	179,714	341.2

*Time period from July through June of the following year.

Additional exploratory analyses were performed. We explored whether younger recruits (representing newly enlisted personnel) had a higher incidence of norovirus by building separate models for <20 year-old and ≥40 year-old age groups in the AD population. In addition, weekly counts of encounters were collapsed into monthly counts, and models were refit to assess the sensitivity of the temporal grouping. To visualize any seasonal patterns among different age groups, norovirus-attributable encounters were averaged by the week number of the seasonal year over each population’s respective study period, and plotted using a 5-week moving average. Finally, we investigated age-related temporal differences in predicted norovirus activity by calculating lagged correlation coefficients among the predicted norovirus time-series DB age groups; lagged correlations coefficients were also calculated between AD and DB populations.

## Results

**Fig 1:**
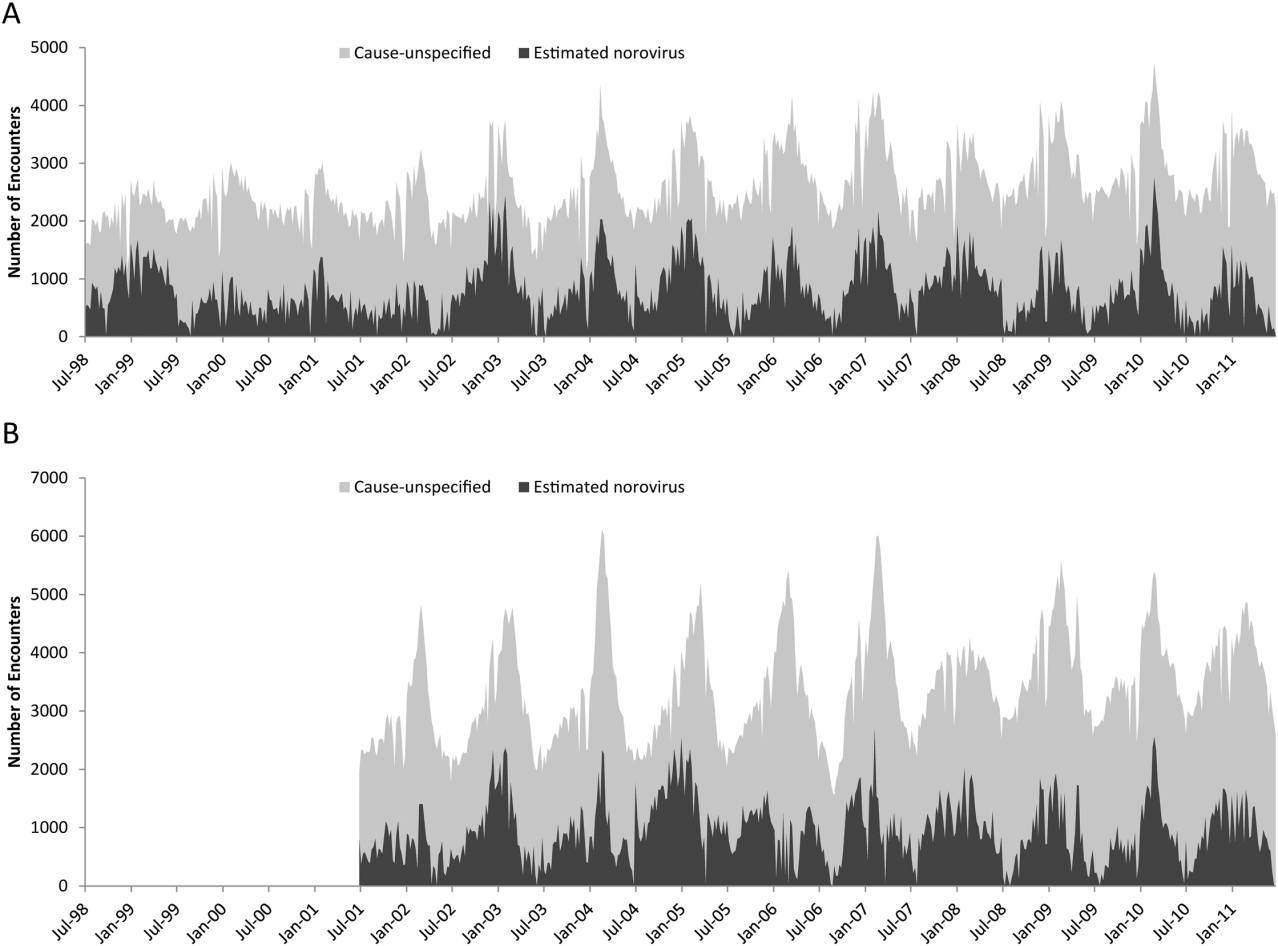
Cause-unspecified and estimated norovirus-attributable encounters by week. (A) Cause-unspecified and estimated norovirus-attributable encounters by week among US military active duty members, July 1998–June 2011. (B) Cause-unspecified and estimated norovirus-attributable encounters by week among dependent beneficiaries of US military active duty members, July 2001–June 2011.

Between July 1998 and June 2011, there were 1,758,562 gastroenteritis medical encounters among the AD population, for a mean of 135,274 visits per seasonal year (Fig 1; Table 2). Among the DB population, in July 2001–June 2011, there were 1,797,140 gastroenteritis medical encounters, for a mean of 179,714 visits per seasonal year. The great majority of these were outpatient encounters, with 99.1% and 98.0% among the AD and DB populations, respectively. Among beneficiaries presenting with all-cause gastroenteritis, over 96% were less than 40 years of age, with 54% in the 0–4 year age group (Table 3). Denominator data used to calculate rates were stable over the study periods, with AD and DB enrollment ranging from ~1,390,000–1,470,000 and ~5,160,000–5,400,000 persons per seasonal year, respectively.

**Table 3:** All-cause, cause-unspecified (CU) and norovirus-attributable encounters by age group.

Population	Seasonal years*	Age Group, Years	All-Cause Encounters, N (% of All Ages)	CU Encounters, N (% of All Ages)	Norovirus-attributable Encounters, N (% of All Ages) †	Norovirus-attributable/CU, %
Active Duty	July 1998–June 2011	All Ages	1,758,562 (100)	1,732,903 (100)	540,377 (100)	31.2
Dependent Beneficiary	July 2001–June 2011	0–4	969,093 (53.9)	952,063 (53.9)	254,925 (52.1)	26.8
Dependent Beneficiary	July 2001–June 2011	5–17	326,047 (18.1)	320,984 (18.2)	173,786 (35.5)	54.1
Dependent Beneficiary	July 2001–June 2011	18–39	440,520 (24.5)	432,144 (24.5)	116,303 (23.8)	26.9
Dependent Beneficiary	July 2001–June 2011	40–64	60,945 (3.4)	59,119 (3.3)	16,905 (3.5)	28.6
Dependent Beneficiary	July 2001–June 2011	All Ages	1,797,140 (100)	1,764,784 (100)	489,381 (100)	27.7

*Time period from July through June of the following year.

^†^Age-specific dependent beneficiary estimates were generated by separate models and do not sum to the All Ages estimate.

The bacterial category was significantly associated with cause-unspecified gastroenteritis in all models (see Table A in S1 File for model coefficients). The protozoal category was not significant in three models, and three models yielded a protozoal coefficient that was negative. The rotavirus category was significant in all models except in the 18–39 year age group; the 40–64 year age group model yielded a rotavirus coefficient that was negative. Since the coefficients of these protozoal and/or rotavirus variables were small in the models in which they were either negative or non-significant, removing them did not affect estimated norovirus encounters by more than 3% over the entire study periods. As a result, protozoal and rotavirus variables were retained to maintain a consistent approach across all models.

**Fig 2:**
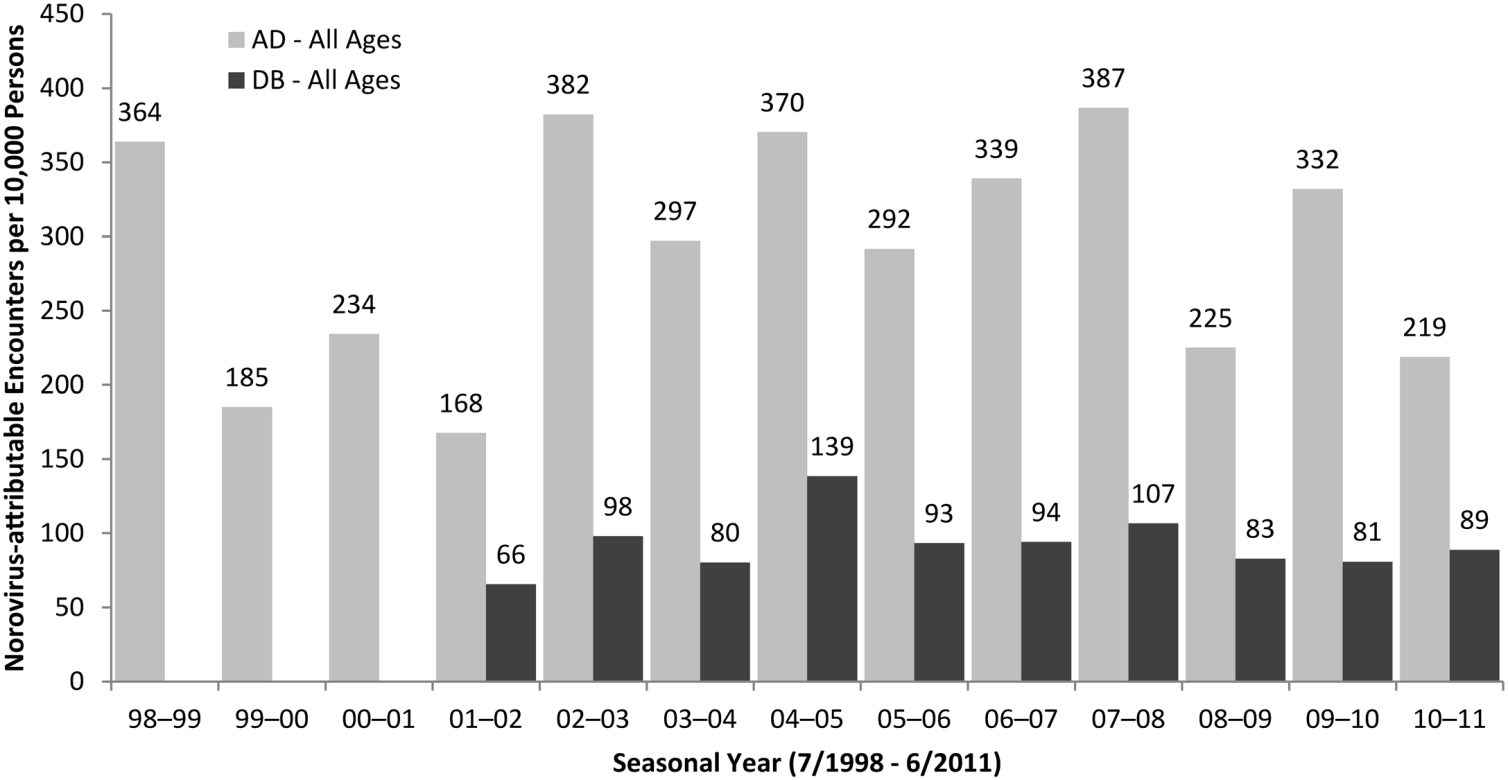
Norovirus-attributable encounters per 10,000 US military active duty (AD) members and their dependent beneficiaries (DB) by seasonal year, July 1998–June 2011.

**Fig 3:**
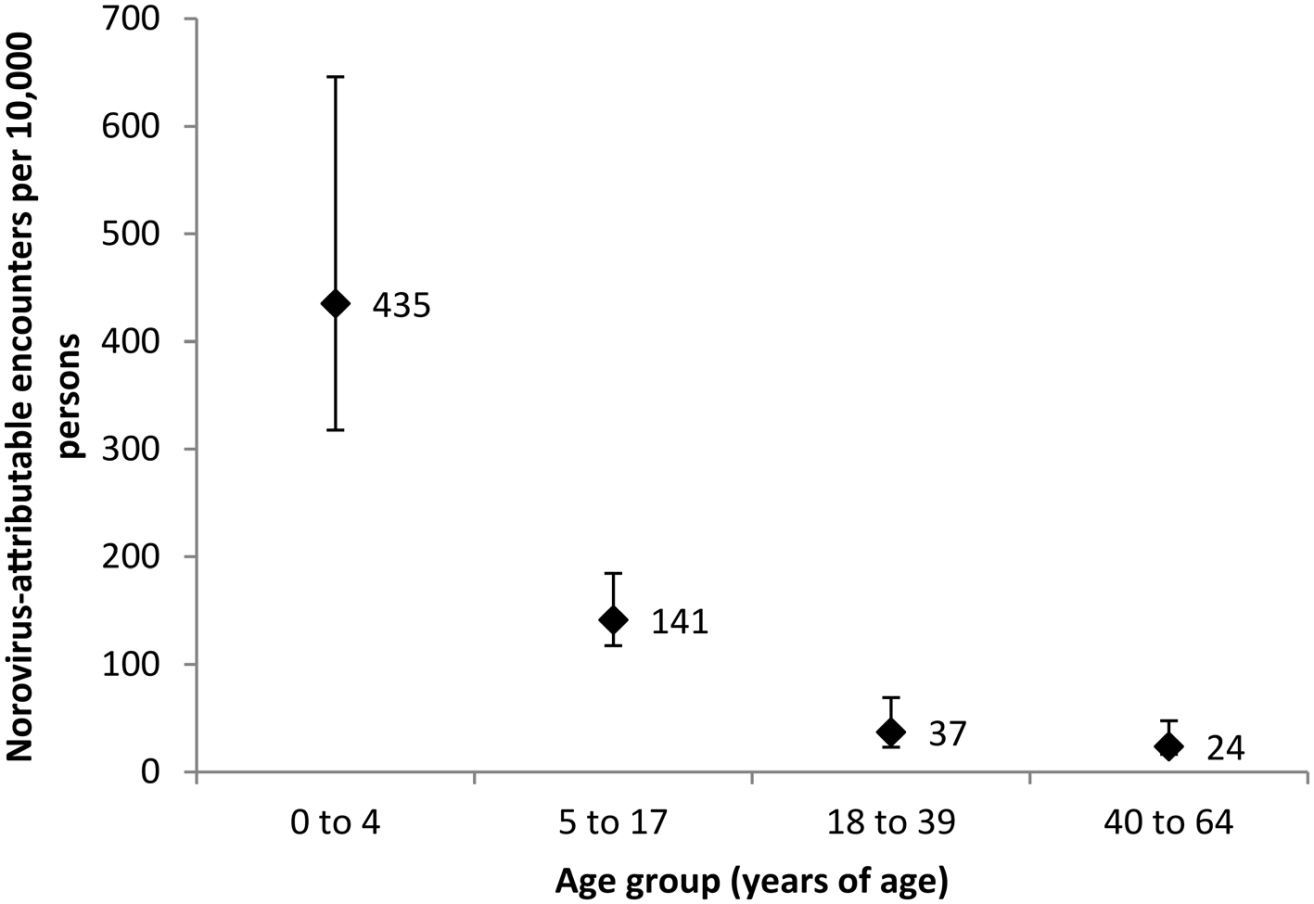
Median seasonal year norovirus-attributable encounters per 10,000 beneficiary persons by age group (with ranges), July 2001–June 2011.

Weekly estimates of norovirus visits exhibited winter seasonality in both AD and DB populations (Fig 1). For the AD population, we estimated ~540,000 cause-unspecified encounters were attributable to norovirus over 13 seasonal years (July 1998–June 2011), corresponding to a mean of 292 (95% CI: 258 to 326) visits per 10,000 persons, ranging from 168–387 encounters per 10,000 persons by seasonal year (Tables 3 & 4, Fig 2). For the DB population, we estimated ~490,000 cause-unspecified coded encounters were attributable to norovirus over 10 seasonal years (July 2001–June 2011), corresponding to a mean of 93 (95% CI: 80 to 105) visits per 10,000 persons, ranging from 66–139 encounters per 10,000 persons by seasonal year. Estimated norovirus-attributable visits accounted for 30.7% and 27.2% of all-cause gastroenteritis encounters in the AD and DP all ages populations, respectively, with over 60% occurring in from November to April in both the AD (66.6%) and DB all ages (61.6%) populations. The majority of the estimated norovirus visits in the beneficiary population occurred in the 0–4 age group (>50%; Table 3), with seasonal estimates ranging from 318 to 646 visits per 10,000 children with a median of 435 visits per 10,000 children (Fig 3). Norovirus-attributable encounters accounted for 26.8–31.2% of cause-unspecified encounters among all age groups and populations except for the 5–17 year age DB group, where it accounted for 54.1% of cause-unspecified encounters (Table 3).

**Table 4:** Norovirus-attributable encounters by season and population.

Seasonal Year*	Active Duty Encounters		Dependent Beneficiary Encounters (All Ages)	
	N† (95% CI‡)	per 10,000 Persons (95% CI)	N† (95% CI‡)	per 10,000 Persons (95% CI)
98–99	51,200 (45,700–56,700)	364 (325–403)		
99–00	25,800 (21,200–30,400)	185 (152–218)		
00–01	32,700 (28,200–37,300)	234 (202–267)		
01–02	23,700 (18,200–29,200)	168 (129–207)	34,000 (27,400–40,500)	66 (53–79)
02–03	55,100 (50,900–59,400)	382 (353–412)	52,000 (45,200–58,800)	98 (85–111)
03–04	43,200 (38,800–47,600)	297 (267–327)	42,900 (36,200–49,600)	80 (68–93)
04–05	53,200 (48,400–57,900)	370 (338–403)	72,800 (66,200–79,400)	139 (126–151)
05–06	41,100 (36,500–45,600)	292 (259–324)	48,300 (41,500–55,200)	93 (80–106)
06–07	47,600 (43,000–52,200)	339 (306–372)	48,600 (41,800–55,500)	94 (81–107)
07–08	54,300 (49,300–59,200)	387 (352–422)	55,400 (49,600–61,300)	107 (95–118)
08–09	32,300 (27,200–37,300)	225 (190–260)	43,900 (37,400–50,300)	83 (71–95)
09–10	48,300 (43,200–53,300)	332 (297–367)	43,400 (36,900–50,000)	81 (69–93)
10–11	32,000 (26,400–37,500)	219 (181–257)	48,000 (41,000–55,000)	89 (76–102)
**Yearly Average**	**41,600 (36,700–46,400)**	**292 (258–326)**	**48,900 (42,300–55,600)**	**93 (80–105)**

*Time period from July through June of the following year.

^†^Number of visits rounded to nearest 100.

^‡^Seasonal 95% confidence intervals calculated by summing up weekly lower and upper bound estimates by season.

Additional exploratory analyses were performed aiming to identify drivers of incidence. Based on models using monthly (rather than weekly) data, we estimated means of 226 (95% CI: 162 to 290) and 71 (95% CI: 51 to 90) norovirus encounters per 10,000 members of the AD and DB populations, respectively. This constituted 23% and 24% fewer norovirus-attributable encounters in the AD population and DB population, respectively, compared to estimates derived from weekly models. Next, models were run separately for weekly data from active duty members who were <20 years old and ≥40 years old. Throughout the study period, rates of estimated norovirus visits in the <20 year-old age group (seasonal year range: 248–437 encounters per 10,000 persons) were consistently higher than those in the ≥40 year-old age group (seasonal year range: 116–229 encounters per 10,000 persons).

**Fig 4:**
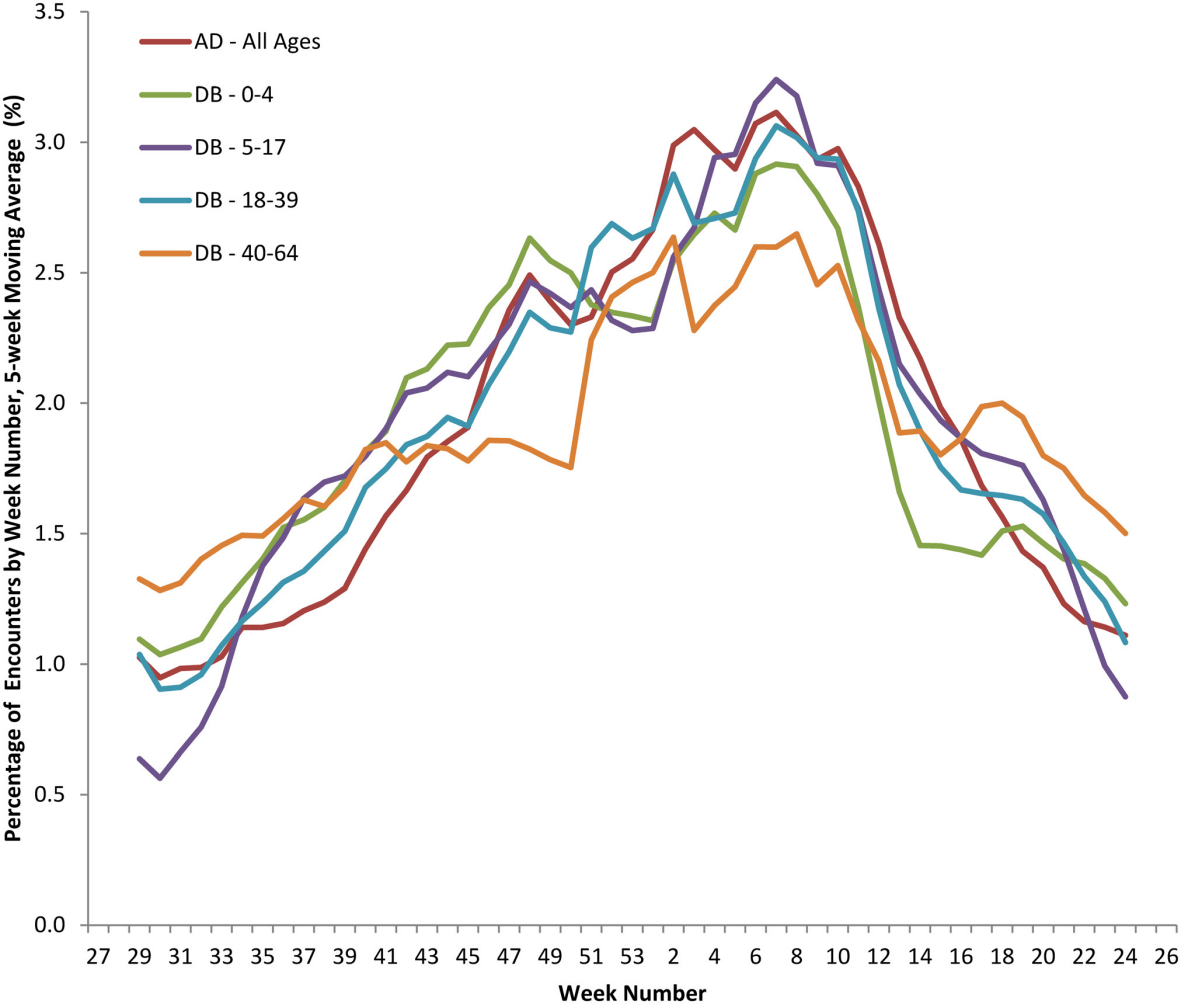
Weekly percent of total norovirus-attributable medical encounters; 5-week moving average by week of year and population. The percentage of estimated norovirus-attributable medical encounters for each week of the year were calculated for each population/age group as follows: the number of encounters occurring in a given week was averaged over the entire study period for each respective group, then expressed as a percentage of the sum of all weekly averages for that population/age group. Five-week moving averages of these values were calculated and plotted for each population/age group. AD: Active duty; DB: Dependent beneficiary; age group range in years.

Finally, estimates of norovirus-attributable encounters were averaged by the week number of the seasonal year over each population’s respective study period and plotted using a 5-week moving average (Fig 4). Norovirus encounters exhibited a winter seasonality that was consistent across populations and age groups. The strongest correlations were for un-lagged data: norovirus activity was synchronized across the age groups (Table B in S1 File).

## Discussion

We estimated 292 and 93 norovirus-attributable encounters per 10,000 persons per seasonal year for the AD and DB populations, respectively, corresponding to an annual estimate of ~42,000 and ~49,000 AD and DB encounters, respectively. Norovirus-attributable encounters represent 31% and 27% of all-cause gastroenteritis visits in the AD and DB populations, respectively, highlighting the importance of norovirus as a cause of gastroenteritis in these settings. Our estimates are consistent with what is known about norovirus epidemiology, with peak of activity occurring in the winter [2, 17, 23–25]. In addition, rates were substantially higher among children <5 years of age compared to other age groups, even when accounting for rotavirus-coded visits [4, 7, 26].

While sharing these general features with non-DoD population-based studies, our estimates for medically-attended norovirus were higher than rates reported previously [27], including the 64 (90% CI: 36–120) norovirus visits per 10,000 persons based on selective testing of members of a health maintenance organization in the state of Georgia in 2004–05 [2] or the 57 (95% CI: 40–74) per 10,000 persons in the US in 2001–09 based on a similar modeling approach using national insurance claim databases, in which the proportion of cause-unspecified encounters (95–97%) were similar to that seen in our population [7]. Studies in European populations have produced comparable estimates ranging from 21 (95% CI: 14–30) to 92 (95% CI: 50–150) outpatient visits per 10,000 persons [4, 26, 28, 29]. Our estimate of 435 norovirus visits per seasonal year among children <5 years of age were also higher than previous civilian estimates based on active surveillance of children <5 years of age in three US counties (319 visits per 10,000 children <5 years of age) [17], extrapolation from a systematic review (167 outpatient visits per 10,000 children) [30] or statistical regression modeling (233 outpatient visits per 10,000 children [95% CI: 156–310]) [7].

We considered several hypotheses as to why our estimates were higher than previous reports in civilian populations. First, our assumption that all residual seasonality was due to norovirus may have overestimated the number of norovirus encounters since there are other viral etiologies of acute gastroenteritis that have winter seasonality such as astrovirus, sapovirus, and rotavirus. However, astrovirus and sapovirus occur at substantially lower levels than norovirus, as does adenovirus [2, 3, 31]. We accounted for rotavirus in all models as it is a major cause of gastroenteritis, particularly in those <5 years of age. Although rotavirus-coded encounters have been shown to only comprise a small minority of viral encounters in the active duty population [32], our modeling approach attributed a substantial fraction of acute gastroenteritis to rotavirus in all age groups except in adult dependent beneficiaries. When explicitly accounting for rotavirus in our model, no appreciable decline in estimated norovirus encounters was observed after 2006, the year rotavirus vaccine was introduced in the US.

Another factor potentially contributing to overestimation was the use of weekly count data, in contrast to monthly intervals used by Gastañaduy et al [7]. It was expected that weekly data would lead to greater variability in the model residuals, yielding a minimum residual of lower magnitude than would be expected using monthly data with less variability; as a result, our estimates based on weekly data would be expected to be higher. We observed this phenomenon in a sensitivity analysis in which collapsing weekly data into months yielded lower estimates. Although the revised estimates for the DB population more closely approximated previously reported estimates [2, 7], the revised estimate for the AD population remained markedly higher.

Given that these methodological biases may not completely explain higher rates of medically-attended norovirus, especially among the active duty military population, it remains possible that active duty personnel and their beneficiaries genuinely have greater care-seeking and/or disease risk. Compared to the general civilian population, DoD service members and their dependent families have increased access to high quality free medical care, likely leading to increased utilization for a range of health concerns [33]. That our estimates for medically-attended norovirus were lower than community incidence estimates for the US civilian population (ranging from 650 [90% CI: 370–1200] [2] to 698 [90% CI: 430–1028] norovirus illnesses per 10,000 persons [5]) suggests greater care-seeking as a plausible explanation. Military personnel may also be subject to higher disease rates as they are more likely to work and live in close quarters and shared common spaces, factors that facilitate norovirus transmission. We attempted to assess this by comparing incidence of norovirus-attributable encounters between the <20 year-old age group and the ≥40 year-old age group. We hypothesized that the rates of norovirus visits would be higher among those <20 years of age who, as lower-ranked members, are more likely to live and work with others in shared confined settings. In contrast, those ≥40 years of age are more likely to be more senior in rank and comparatively spend less time in shared confined spaces. We found that rates of estimated norovirus visits among those <20 years old were consistently higher than those ≥40 years old throughout the study period, supporting this hypothesis.

Other limitations of our study should be considered in addition to the considerations for overestimation mentioned above. The statistical method assumes that coding and testing practices did not change over time. We addressed this possibility by adding a time variable to account for any secular changes over the study period. In addition, our analyses used counts of diagnostic codes to provide estimates that assumed one diagnostic code per visit. However, we could not account for the possibility of medical encounters that had more than one associated code, which may have also led to overestimation. Next, a minority (<15%) of all coded medical encounters were known to occur outside of the continental US (data not shown). In principle, their inclusion could have resulted in a dilution of the seasonality of AGE. However, model residuals still exhibited a clear wintertime seasonality consistent with norovirus activity observed in the continental US (Fig 1). Underestimation of norovirus disease was also possible, as we assumed that no norovirus visits occurred in one week of the year, whereas norovirus is known to circulate year-round [2, 17, 25]. Finally, although we found no evidence of temporal variability in norovirus activity across age groups suggesting that one age group spread infection to another, our study did not evaluate questions such as service-specific rates of norovirus disease, spatiotemporal dynamics of norovirus disease, or specifying illnesses in deployed populations. In particular, deployed personnel could not be differentiated from non-deployed personnel in our model due to the aggregate nature of both the medical encounter and total enrollment data. Additional studies may help better evaluate the impact norovirus disease has on cost, lost productivity, and potential mission impact among US DoD populations.

While the full impact of norovirus disease on the US military is still to be quantified, our results highlight the importance of norovirus as a cause of infectious gastroenteritis among active duty military personnel and their beneficiaries in non-deployment settings. Active surveillance studies using sensitive clinical diagnostics for norovirus would help further solidify estimates of the burden of disease in military populations. Risk factor studies among AD and DB could improve our understanding of the epidemiology of norovirus and elucidate why rates are apparently higher in these populations. Finally, our results point to the potential benefits of vaccines, which are currently in development, and establish baseline estimates with which to evaluate the value of such an intervention [34, 35].

## Notes

1. The views expressed in this article are those of the authors and do not necessarily reflect the official policy or position of the Department of the Navy, Department of Defense, nor the U.S. Government.
2. Two of the authors (MR and CP) are military service members and employees of the U.S. Government. This work was prepared as part of their official duties. Title 17 U.S.C. §105 provides that ‘Copyright protection under this title is not available for any work of the United States Government.’ Title 17 U.S.C. §101 defines a U.S. Government work as a work prepared by a military service member or employee of the U.S. Government as part of that person’s official duties.
3. Results of preliminary analysis of this data was presented as an abstract at the Vaccines for Enteric Diseases (VED 2015) Conference in Edinburgh, UK, July 8–10, 2015.

## Supporting Information

S1 File Supplemental Tables. Model parameter estimates (**Table A**). Lagged correlation coefficients between the estimated norovirus time-series among populations and age groups (**Table B**).
(DOCX)
