# Supplementary material for: Incidence of Norovirus-Associated Medical Encounters among Active Duty United States Military Personnel and Their Dependents
Source: PLoS One. 2016 Apr 26;11(4):e0148505. doi: 10.1371/journal.pone.0148505 (PMC4845987; doi:10.1371/journal.pone.0148505)
Supplement: S1 File — Model parameter estimates (Table A). Lagged correlation coefficients between the estimated norovirus time-series among populations and age groups (Table B). (DOCX) [file pone.0148505.s001.docx]

**S1 File. Supplemental Tables**

**Table A**: Model parameter estimates.

| Population | Seasonal Years* | Age Group | Intercept | | | Bacterial | | | Protozoal | | | Rotavirus | | | Time | | |
| --- | --- | --- | --- | --- | --- | --- | --- | --- | --- | --- | --- | --- | --- | --- | --- | --- | --- |
|  |  |  | α | 95% CI | *P*-value† | β_1_ | 95% CI | *P*-value† | β_2_ | 95% CI | *P*-value† | β_3_ | 95% CI | *P*-value† | γ | 95% CI | *P*-value† |
| Active Duty | July 1998–June 2011 | All Ages | 1895.7 | 1779.1 to 2012.2 | <0.01 | 10.6 | 6.4 to 14.8 | <0.01 | 2.3 | -15.3 to 20.0 | 0.79 | 87.6 | 50.4 to 124.7 | <0.01 | 1.0 | 0.8 to 1.2 | <0.01 |
| Dependent Beneficiary | July 2001–June 2011 | 0–4 | 1036.8 | 944.4 to 1129.3 | <0.01 | 5.6 | 0.7 to 10.4 | 0.03 | 12.0 | -1.3 to 25.3 | 0.08 | 24.5 | 22.5 to 26.5 | <0.01 | 0.8 | 0.6 to 1.0 | <0.01 |
|  |  | 5–17 | 438.7 | 381.2 to 496.2 | <0.01 | 5.5 | 1.4 to 9.7 | 0.01 | -20.1 | -31.8 to -8.5 | <0.01 | 50.8 | 37.7 to 63.9 | <0.01 | 0.3 | 0.2 to 0.4 | <0.01 |
|  |  | 18–39 | 443.3 | 412.0 to 474.5 | <0.01 | 3.9 | 1.3 to 6.6 | <0.01 | -6.9 | -13.3 to -0.4 | 0.04 | 18.9 | -10.5 to 48.3 | 0.21 | 0.8 | 0.7 to 0.9 | <0.01 |
|  |  | 40–64 | 63.7 | 60.0 to 67.3 | <0.01 | 0.7 | 0.0 to 1.3 | 0.04 | 2.6 | 0.1 to 5.1 | 0.04 | -5.9 | -11.8 to -0.1 | 0.05 | 0.1 | 0.1 to 0.1 | <0.01 |
|  |  | All Ages | 1709.1 | 1475.9 to 1942.3 | <0.01 | 10.5 | 4.7 to 16.3 | <0.01 | -0.3 | -15.9 to 15.3 | 0.97 | 33.0 | 29.9 to 36.1 | <0.01 | 1.9 | 1.5 to 2.2 | <0.01 |

*****Time period from July through June of the following year.

†*P*-values based on Z-test.

**Table B**: Lagged correlation coefficients between the estimated norovirus time-series among populations and age groups.

|  |  | **Active Duty** | **Dependent Beneficiary** | | | | |
| --- | --- | --- | --- | --- | --- | --- | --- |
|  | **Age Group (years)** | All Ages | 0–4 | 5–17 | 18–39 | 40–64 | All Ages |
| **Active Duty** | All Ages | 1 |  |  |  |  |  |
| **Dependent Beneficiary** | 0–4 |  | 1 |  |  |  |  |
|  | 5–17 |  | 0.660 | 1 |  |  |  |
|  | 18–39 |  | 0.624 | 0.716 | 1 |  |  |
|  | 40–64 |  | 0.334 | 0.475 | 0.636 | 1 |  |
|  | All Ages | 0.733 |  |  |  |  | 1 |

*Maximum R always occurred with 0 lag.
